# Supplementary material for: Proposal for the Inclusion of Tobacco Use in Suicide Risk Scales: Results of a Meta-Analysis
Source: Int J Environ Res Public Health. 2021 Jun 5;18(11):6103. doi: 10.3390/ijerph18116103 (PMC8201119; doi:10.3390/ijerph18116103)
Supplement: Supplementary file 1 [file ijerph-18-06103-s001.zip › Supplementary Materials.pdf]

(a) Former Smokers:

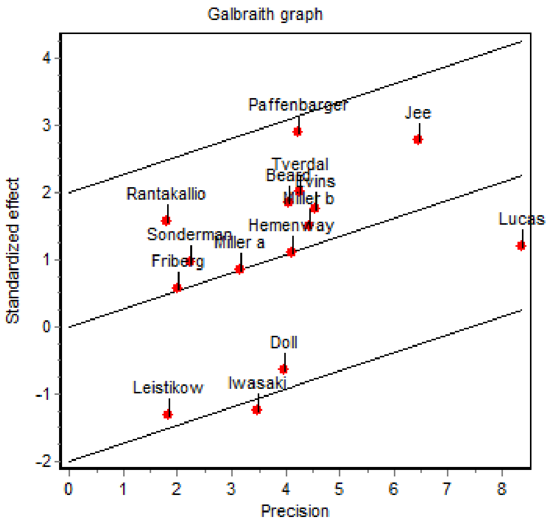

(b) Current Smokers:

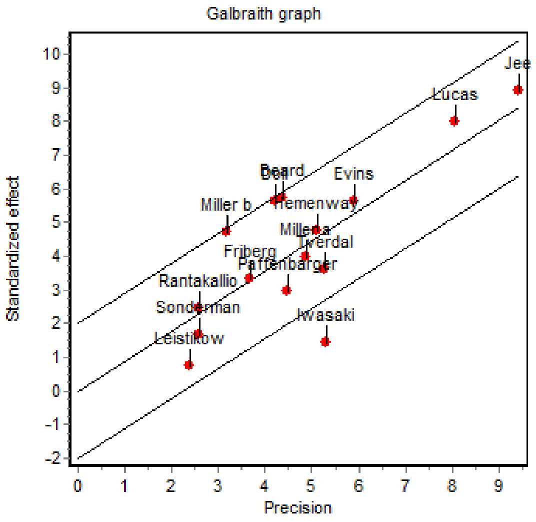

(c) Smoker Women:

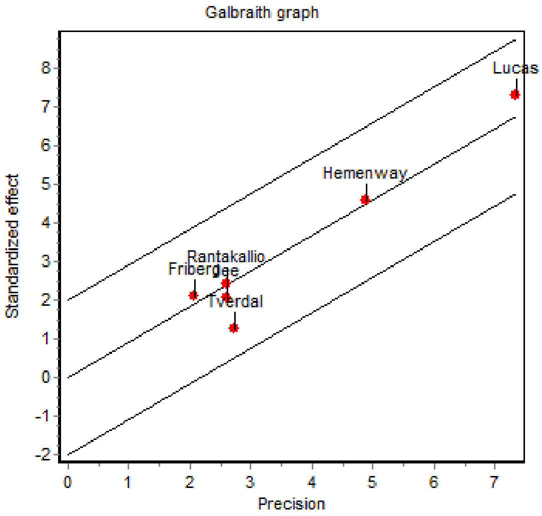

(d) Smoker Men:

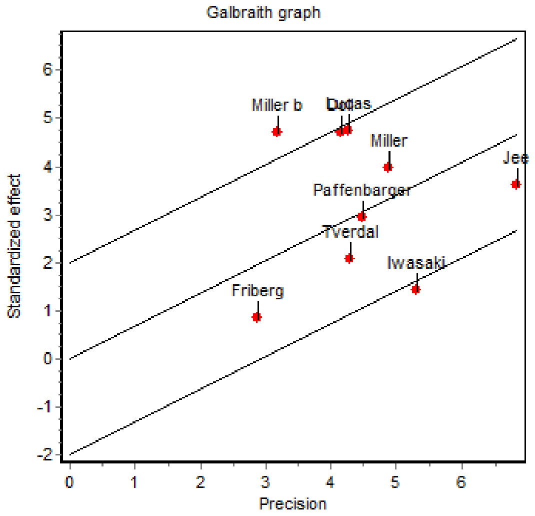

Figure S1. Galbraith graphs of suicide in former, current, female, and male smokers.

(a) Former Smokers Ideation:

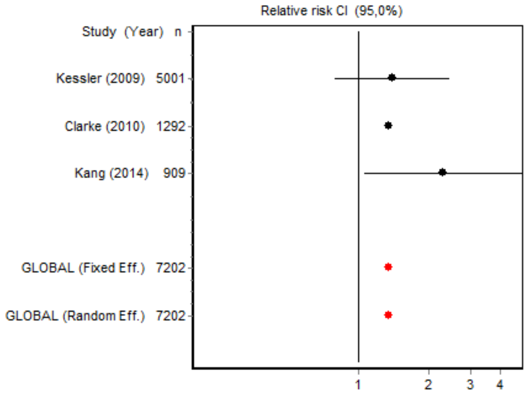

(b) Current Smokers Ideation:

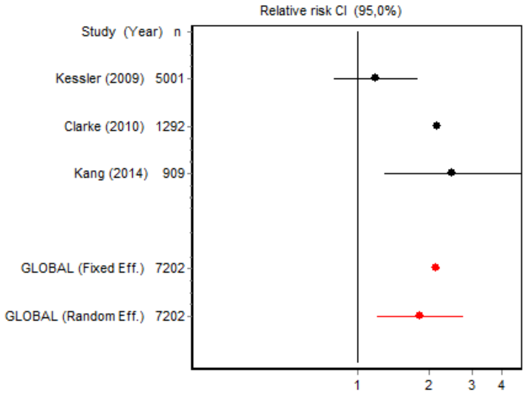

(c) Former Smokers Attempts:

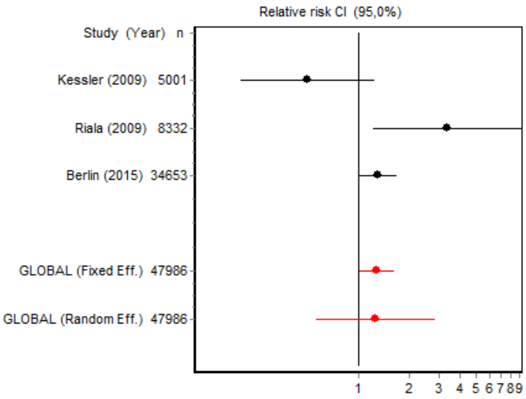

(d) Current Smokers Attempts:

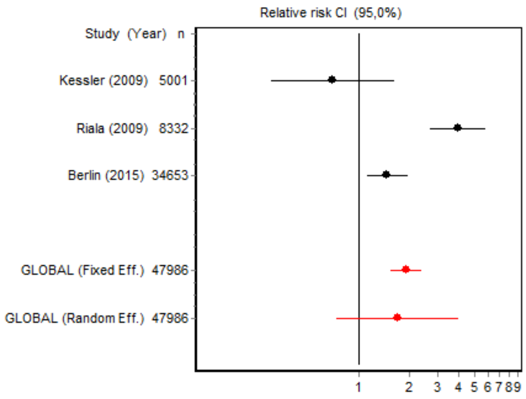

Figure S2. Forest Plots of suicidal ideation and attempts in former and current smokers.

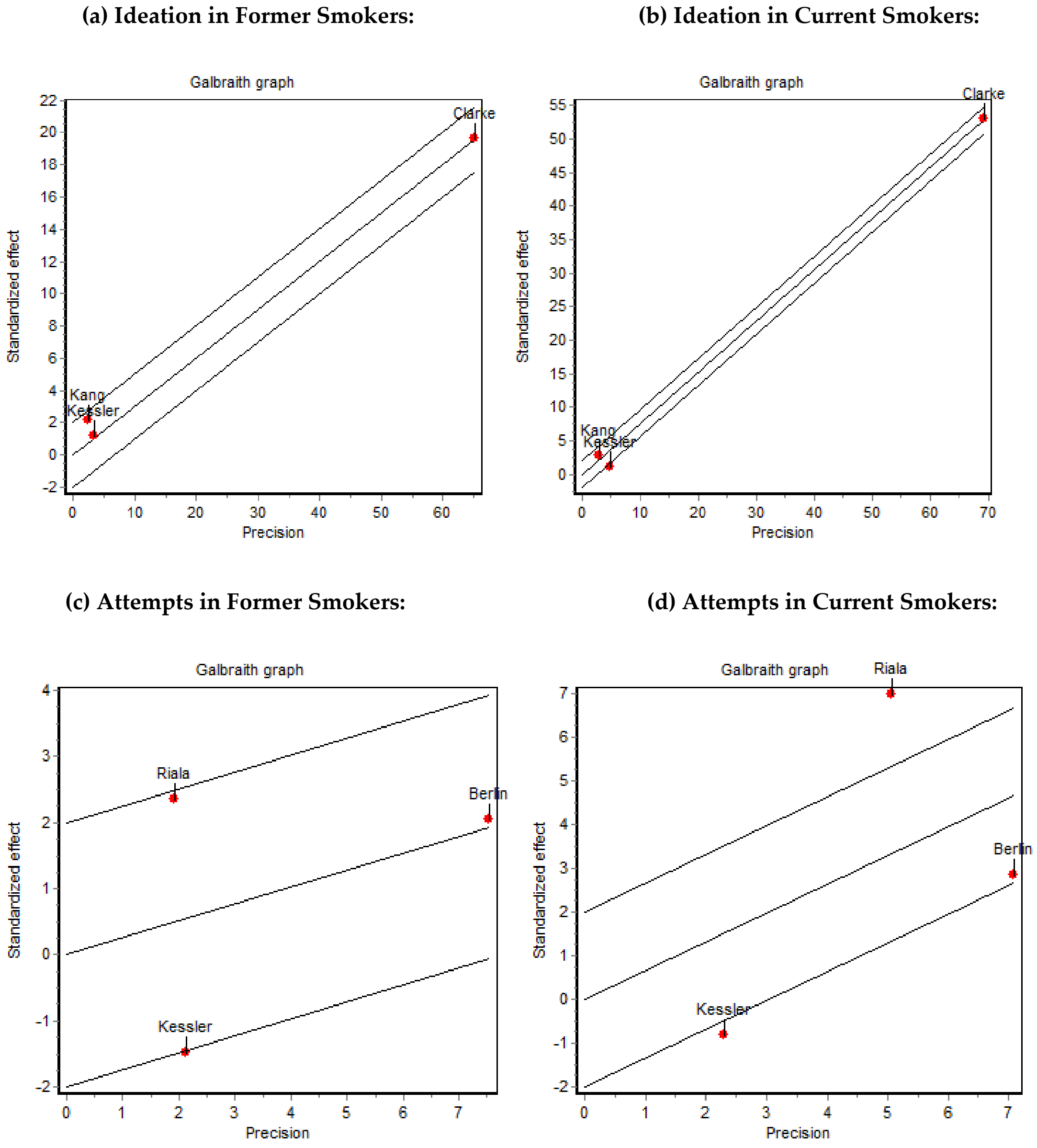

Figure S3. Galbraith graphs of suicidal ideation and attempts in former and current smokers.

(a) First meta-analysis: **(b) Meta-analysis without Clarke (2010):**

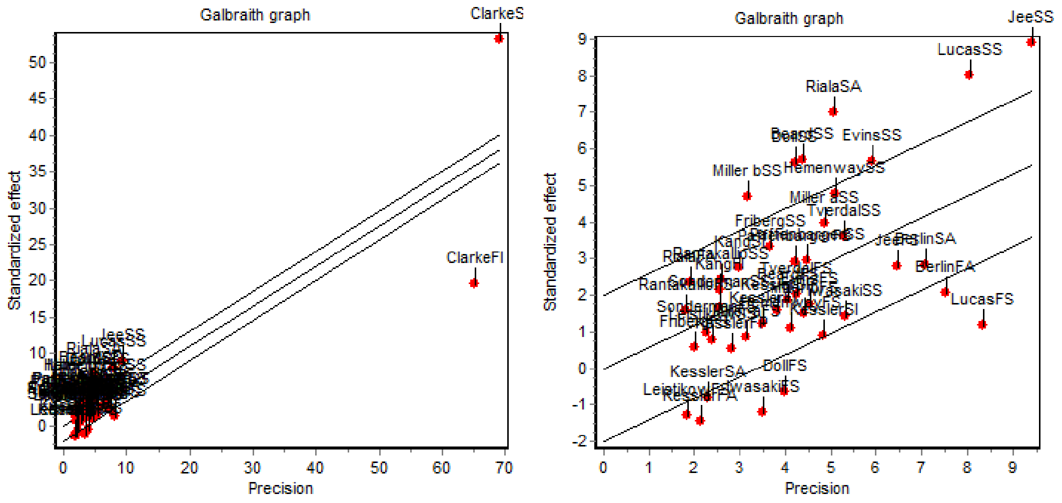

Figure S4. Galbraith graphs of all suicidal behaviours in former and current smokers.

(a) Ideation in Former Smokers:

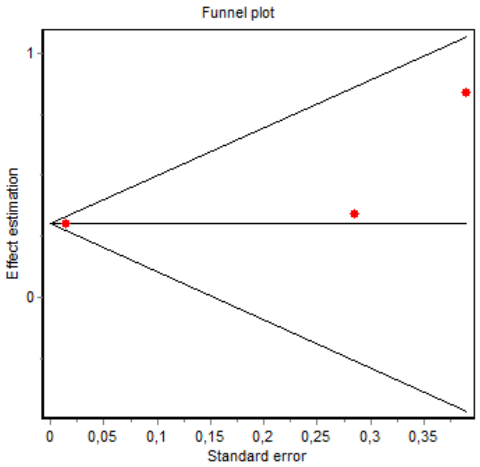

(b) Ideation in Current Smokers:

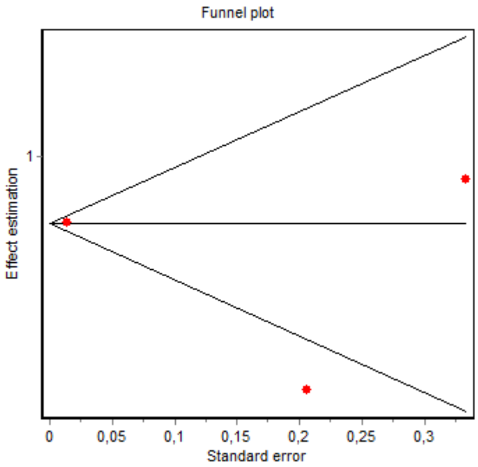

(c) Attempts in Former Smokers:

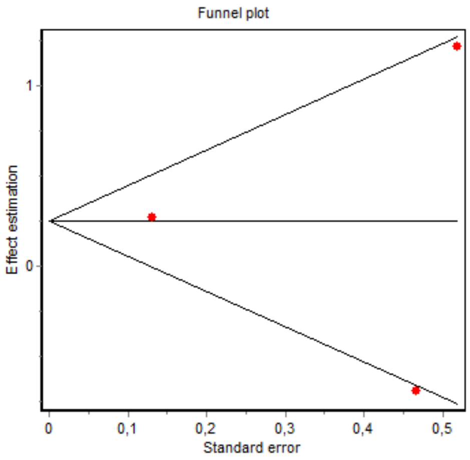

(d) Attempts in Current Smokers:

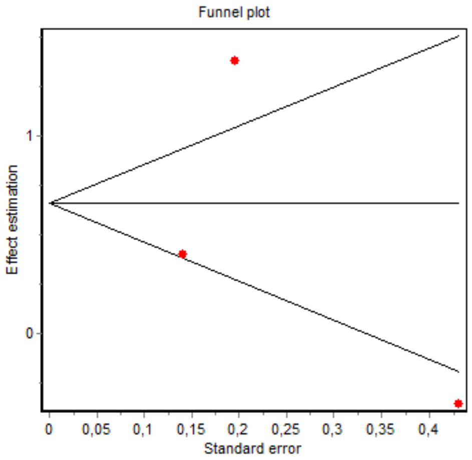

Figure S5. Funnel plots of suicidal ideation and attempts in former and current smokers.

(a) Former Smokers:

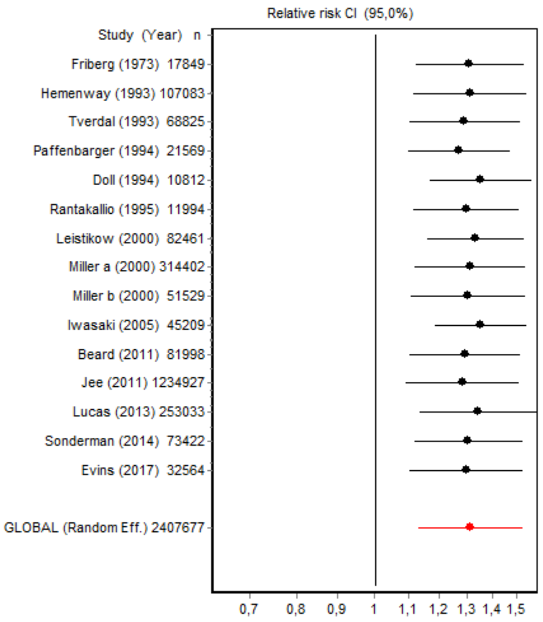

(b) Current Smokers:

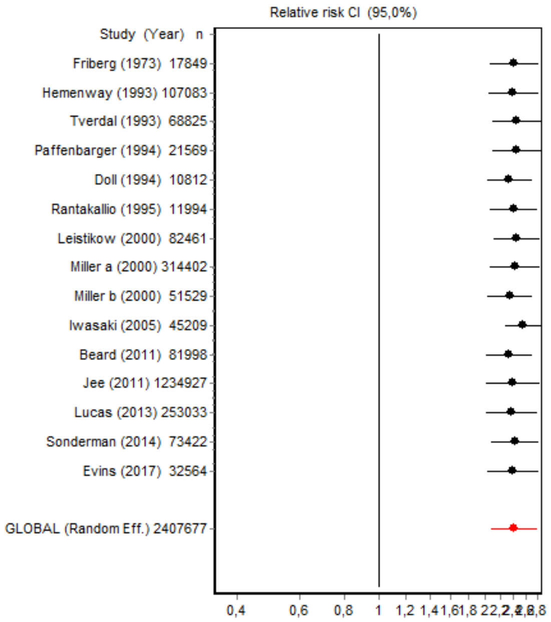

(c) Smoker Women:

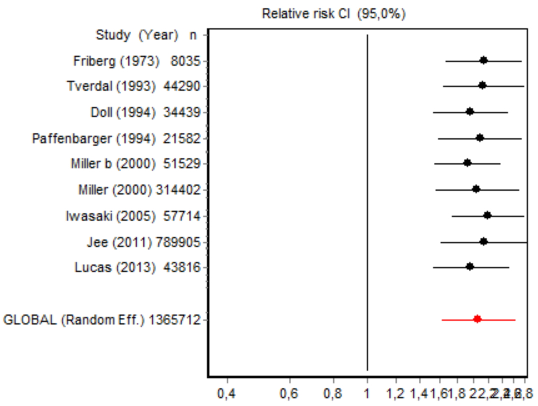

(d) Smoker Men:

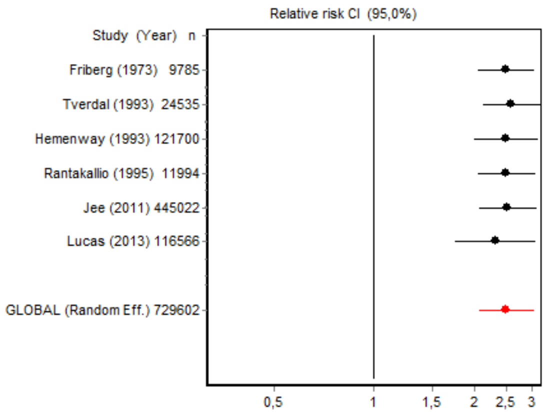

Figure S6. Influence graphs of suicide in former, current, female, and male smokers.

(a) Ideation in Former Smokers:

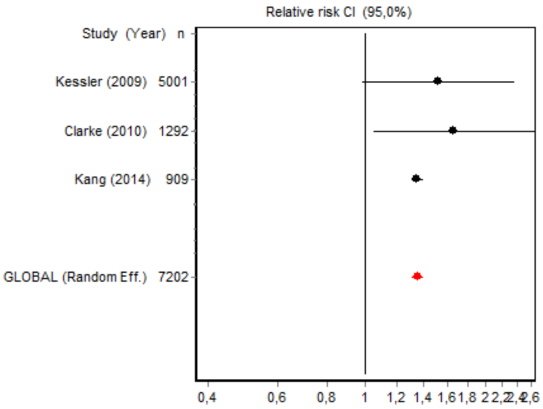

(b) Ideation in Current Smokers:

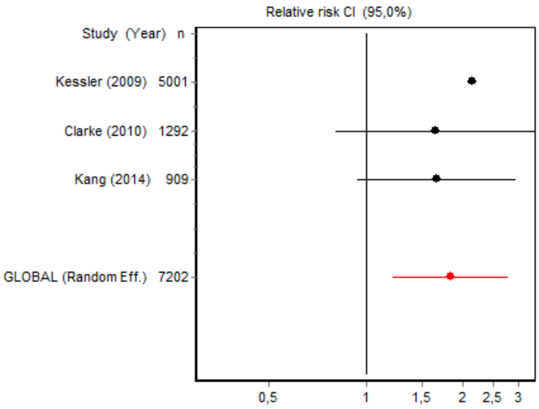

(c) Attempts in Former Smokers:

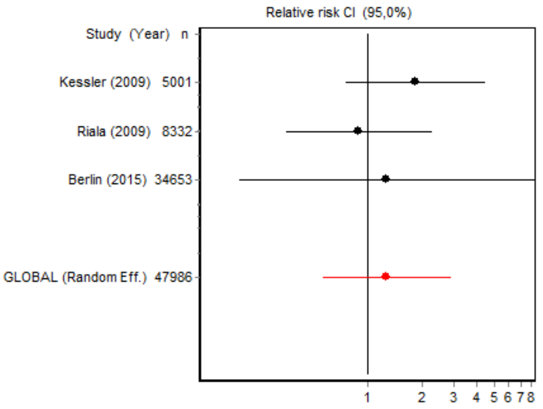

(d) Attempts in Current Smokers:

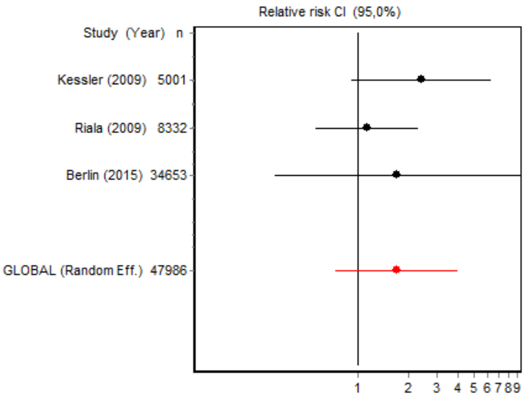

Figure S7. Influence graphs of suicidal ideation and attempts in former and current smokers.

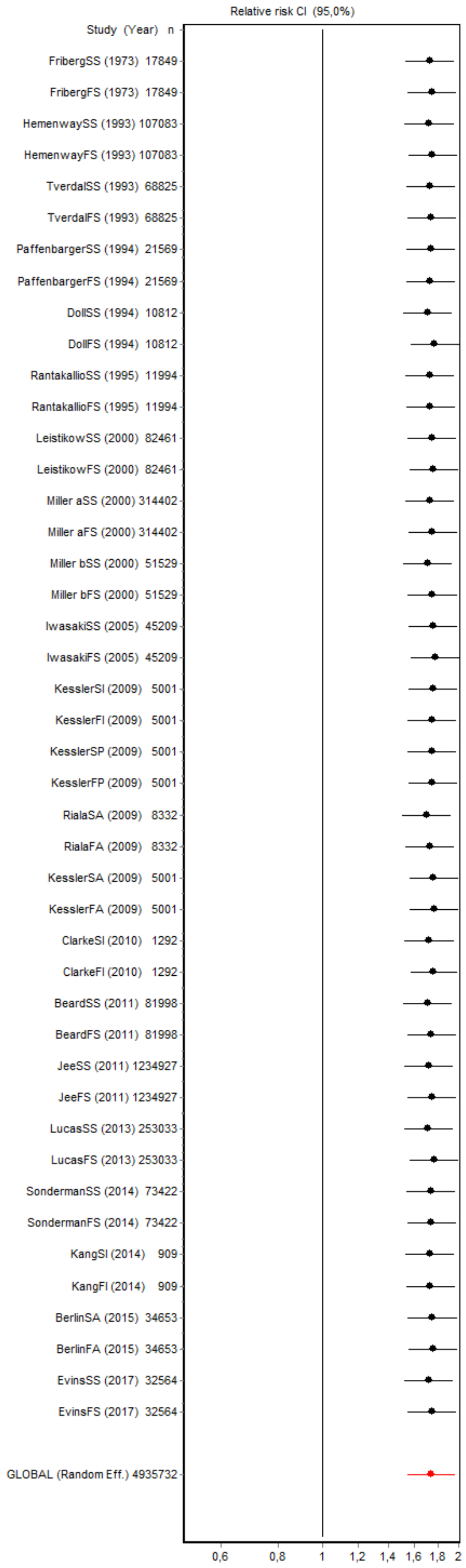

Figure S8. Influence graph of all suicide behaviours in former and current smokers.
